# ERC-ESICM-Leitlinien zur Temperaturkontrolle nach Herz-Kreislauf-Stillstand

**DOI:** 10.1007/s00101-022-01148-1

**Published:** 2022-06-15

**Authors:** Marlene Fischer, Elena Kainz

**Affiliations:** 1 Klinik und Poliklinik für Anästhesiologie, Zentrum für Anästhesiologie und Intensivmedizin, Universitätsklinikum Hamburg-Eppendorf, Martinistr. 52, 20246 Hamburg, Deutschland; 2 Klinik für Intensivmedizin, Zentrum für Anästhesiologie und Intensivmedizin, Universitätsklinikum Hamburg-Eppendorf, Martinistr. 52, 20246 Hamburg, Deutschland

## Abstract

Die Leitlinien des European Resuscitation Council (ERC), die 2021 veröffentlicht wurden, empfehlen eine aktive Temperaturkontrolle zwischen 32 und 36 °C für alle erwachsenen Patient:innen, die nach Wiedererlangen des Spontankreislaufs nach prä- oder innerklinischer Reanimation das Bewusstsein nicht wiedererlangen.

Wenige Wochen nach Veröffentlichung der Leitlinien 2021 wurde die TTM2-Studie publiziert, in der kein signifikanter Unterschied im Hinblick auf das Überleben oder das funktionelle Outcome nach sechs Monaten zwischen einer Zieltemperatur von 33 °C und einer Fiebervermeidung nach außerklinischem Herz-Kreislauf-Stillstand festgestellt worden war.

Als Antwort auf die zusätzliche Evidenz durch die TTM2-Studie nahm die Advanced Life Support Task Force des International Liaison Committee on Resuscitation (ILCOR) eine Reevaluation der aktuellen Datenlage vor, die in einer überarbeiteten Empfehlung zur Temperaturkontrolle nach Herz-Kreislauf-Stillstand bei Erwachsenen resultierte. Der aktuelle Artikel fasst die aktualisierten Leitlinien zusammen und diskutiert kritische Aspekte der neuen Empfehlungen.

## Einleitung

Die Leitlinien des European Resuscitation Council (ERC), die 2021 veröffentlicht wurden, empfehlen eine aktive Temperaturkontrolle für alle erwachsenen Patient:innen, die nach Wiedererlangen des Spontankreislaufs nach prä- oder innerklinischer Reanimation das Bewusstsein nicht wiedererlangen [18]. Gemäß Empfehlung sollte eine Zieltemperatur zwischen 32 und 36 °C angestrebt werden.

Wenige Wochen nach Veröffentlichung der Leitlinien 2021 wurde die TTM2-Studie publiziert, in der eine Temperaturkontrolle mit einer Zieltemperatur von 33 °C mit einer Fiebervermeidung nach außerklinischem Herz-Kreislauf-Stillstand verglichen worden war [7]. Dabei wurde kein signifikanter Unterschied im Hinblick auf das Überleben oder das funktionelle Outcome nach sechs Monaten zwischen den beiden Temperaturregimes festgestellt.

Darüber hinaus untersuchte eine Netzwerk-Metaanalyse den Effekt von Hypothermie (31–36 °C) versus Normothermie (37–37,8 °C) nach außerklinischem Herz-Kreislauf-Stillstand auf Letalität und funktionelles Outcome nach sechs Monaten [9]. In den insgesamt zehn berücksichtigten randomisierten Studien wurde keine protektive Wirkung einer therapeutischen Hypothermie nachgewiesen.

## Aktualisierung der Empfehlungen zur Temperaturkontrolle

Als Antwort auf die zusätzliche Evidenz durch die TTM2-Studie nahm die Advanced Life Support Task Force des International Liaison Committee on Resuscitation (ILCOR) eine Reevaluation der aktuellen Datenlage vor, die in einer überarbeiteten Empfehlung zur Temperaturkontrolle nach Herz-Kreislauf-Stillstand bei Erwachsenen resultierte [10, 21, 22].

**Figure 1:**
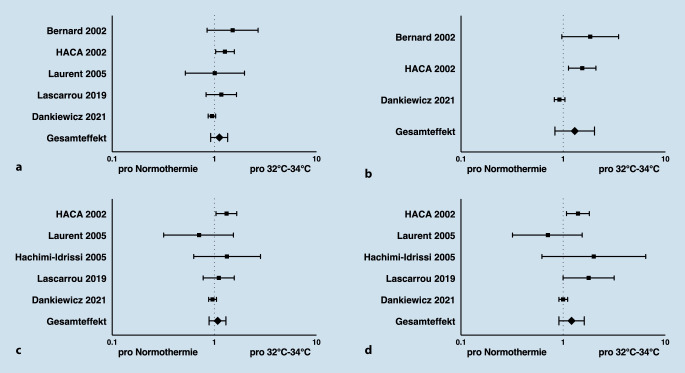

In der Metaanalyse der ILCOR Advanced Life Support Task Force wurden sechs randomisierte Studien berücksichtigt, die eine Zieltemperatur von 32–34 °C für die Dauer von 12–24 h im Vergleich zu Fiebervermeidung/Normothermie untersuchten. Dabei zeigte sich kein Vorteil hinsichtlich des Überlebens (Abb. 1a) oder des funktionellen Outcome (Abb. 1b) bei Entlassung aus dem Krankenhaus oder 30 Tage nach Herz-Kreislauf-Stillstand. Auch 90–180 Tage nach Herz-Kreislauf-Stillstand konnte kein signifikanter Effekt der therapeutischen Hypothermie nachgewiesen werden (Abb. 1c, d).

In derselben Metaanalyse wurden die Ergebnisse von zehn Studien ausgewertet, in der die Anwendung prähospitaler Kühlung mit dem Verzicht auf Kühlmaßnahmen vor Krankenhausaufnahme verglichen worden war. Auch hier konnte kein Vorteil einer präklinischen Hypothermie im Hinblick auf das Überleben und das funktionelle Outcome bei Entlassung aus dem Krankenhaus festgestellt werden.

Die Ergebnisse von insgesamt drei Studien, die endovaskuläre mit Oberflächenkühlung verglichen hatten, wurden ebenfalls in der Metaanalyse der ILCOR berücksichtigt. Zwischen den beiden Kühlmethoden wurde kein Unterschied hinsichtlich des Überlebens oder des neurologischen Outcome bei Entlassung bzw. nach 28 Tagen beobachtet.

Für die Dauer der Temperaturkontrolle ergab sich keine neue Evidenz: Eine randomisierte Studie verglich 24 h mit 48 h Dauer einer Hypothermie von 33 °C nach außerklinischem Herz-Kreislauf-Stillstand, wobei kein signifikanter Unterschied hinsichtlich des Überlebens bzw. des neurologischen Outcome nach sechs Monaten festgestellt wurde [13].

Die beiden genannten Metaanalysen kommen nun zu dem Schluss, dass sich die Anwendung von therapeutischer Hypothermie im Vergleich zu kontrollierter Normothermie/Fiebervermeidung nicht hinsichtlich des Überlebens oder des neurologischen Outcome unterscheidet [9, 22]. Trotz Äquivalenz der beiden Temperaturzielbereiche besteht im Rahmen des ERC-Expertengremiums Konsensus, dass eine Fieberprävention im Vergleich zur therapeutischen Hypothermie unter Umständen ressourcenschonender ist und mit weniger unerwünschten Wirkungen einhergeht. Aus diesem Grund wird in den überarbeiteten ERC-ESICM^1^-Leitlinien einer Normothermie/Fieberprävention gegenüber der Temperaturkontrolle zwischen 32 und 36 °C der Vorzug gegeben. Aus Gründen, die im Anschluss ausführlich diskutiert werden, sprach sich jedoch eine Mehrheit des Expertengremiums für eine Beibehaltung der Temperaturkontrolle von 32–36 °C für ausgewählte Patientenpopulationen aus.

Neben der Wahl der Zieltemperatur nehmen die aktualisierten Leitlinien auch Stellung zu den Aspekten der prähospitalen Kühlung, der Methode und der Dauer der Temperaturkontrolle (Tab. 1). Innerhalb des Expertengremiums herrschte Einigkeit über die Notwendigkeit einer aktiven Temperaturkontrolle mit kontinuierlicher Messung der Körpertemperatur. Die Wahl der Kühl‑/Temperaturkontrollmethode bleibt jedoch dem/r Anwender:in überlassen.

**Table 1:** 

Maßnahme	Grad der Evidenz und Stärke der Empfehlung
Kontinuierliche Messung der Körpertemperatur bei Patient:innen, die nach ROSC komatös bleiben	Good Practice Statement
Aktive Prävention von Fieber (> 37,7 °C)	Schwache Empfehlung, niedrige Qualität der Evidenz
Aktive Prävention von Fieber für mind. 72 h nach Herz-Kreislauf-Stillstand	Good Practice Statement
Temperaturkontrolle durch Antipyretika oder physikalische Maßnahmen, ggf. Geräte-basierte Temperaturkontrolle mit Zieltemperatur 37,5 °C	Good Practice Statement
Keine Empfehlung für oder gegen eine Temperaturkontrolle zwischen 32 und 36 °C oder frühe Hypothermie bei ausgewählten Subpopulationen; keine aktive Wiedererwärmung von Patient:innen mit milder Hypothermie	Good Practice Statement
Empfehlung gegen prähospitale Gabe von hohen Mengen kalter i.v.-Flüssigkeit nach ROSC	Starke Empfehlung, moderate Qualität der Evidenz

*ROSC* „return of spontaneous circulation“ (Wiedererlangen des Spontankreislaufs)

## Diskussion

Aufgrund der Anzahl der eingeschlossenen Patient:innen sind die genannten Metaanalysen und die aktuellen Leitlinien des ERC/ESICM überwiegend durch die TTM-Studien aus 2013 und 2021 gewichtet [7, 16]. Insbesondere die 2021 veröffentlichte TTM2-Studie gab den maßgeblichen Ausschlag für die derzeitige Leitlinienempfehlung [7]. Bei der Interpretation der Ergebnisse der TTM- und insbesondere der TTM2-Studie sowie der Übertragung in den klinischen Alltag gibt es allerdings mehrere relevante Aspekte, die berücksichtigt werden sollten.

### Laienreanimation und No-Flow-Zeit

Ähnlich wie in die TTM-Studie aus 2013 wurden auch in die TTM2-Studie überwiegend Patient:innen mit hohen Ersthelferraten eingeschlossen. Eine hohe Laienreanimationsquote stellt einen der entscheidenden Prädiktoren für ein günstiges neurologisches Outcome dar [25]. In den beiden TTM-Studien wurden Ersthelferraten zwischen 73 und 80 % mit nur sehr geringer No-Flow-Zeit (Median von 1 min in der TTM-Studie) berichtet [7, 16]. Im Tiermodell wurde nachgewiesen, dass eine substanzielle neuronale Schädigung innerhalb von 10–12 min No-Flow-Zeit nach Herz-Kreislauf-Stillstand auftritt [6]. Eine zusätzliche Anwendung neuroprotektiver Maßnahmen bei Patient:innen, bei denen keine relevante hypoxisch-ischämische neuronale Schädigung aufgrund geringer No-Flow-Zeit besteht, erscheint daher nicht zielführend.

Darüber hinaus konnten Böttiger et al. in einer Sekundäranalyse zeigen, dass die Ersthelferrate invers mit einem Vorteil für das neurologische Outcome durch therapeutische Temperaturkontrolle assoziiert ist [3]. Daten von insgesamt acht randomisierten klinischen Studien wurden analysiert. Dabei konnte nachgewiesen werden, dass Populationen mit niedriger Ersthelferrate in einem höheren Ausmaß von therapeutischer Temperaturkontrolle profitierten. Die Autoren schlussfolgern daher, dass eine hohe Ersthelferrate mit geringer No-Flow-Zeit einhergeht und daher der potenziell neuroprotektive Effekt von therapeutischer Temperaturkontrolle bei *per se* geringer oder nicht vorhandener neuronaler Schädigung keine Wirkung erzielen kann.

Die hohen Ersthelferraten und niedrigen No-Flow-Zeiten aus den TTM-Studien sind entscheidende Determinanten für das weitere Überleben und das neurologische Outcome betroffener Patient:innen [25]. Sie spiegeln allerdings die tatsächlichen Zahlen in vielen Ländern nur unzureichend wider [12]. In Deutschland wurde 2020 eine Laienreanimationsquote von 40,4 % beschrieben [8]. Vor dem Hintergrund der vergleichsweise geringen Laienreanimationsquote in Deutschland erscheint der Aspekt der Übertragbarkeit der derzeitigen Evidenz besonders relevant.

Auch innerhalb des Leitliniengremiums sprach sich eine Mehrheit der beteiligten Experten für eine Beibehaltung der Temperaturkontrolle von 32–36 °C für ausgewählte Patientenpopulationen aus. Somit kann diskutiert werden, ob eine differenzierte Temperaturkontrolle für Patient:innen mit niedriger bzw. hoher Laienreanimationsquote und langer bzw. kurzer No-Flow-Zeit erforderlich ist, um die neuroprotektiven Effekte einer therapeutischen Temperaturkontrolle optimal einzusetzen.

### Dauer bis zum Erreichen der Zieltemperatur

In den beiden TTM-Studien betrug die durchschnittliche Zeit bis zum Erreichen der Zieltemperatur 7 h [3]. Tierexperimentelle Daten weisen jedoch darauf hin, dass ein neuroprotektiver Effekt nur dann nachweisbar ist, wenn eine therapeutische Hypothermie innerhalb von 60 min nach Wiedererlangen des Spontankreislaufs eingesetzt wird [1]. Auch dieser Aspekt sollte bei der Interpretation der Studienergebnisse sowie der aktuellen Leitlinienempfehlungen berücksichtigt werden. Unter Umständen sollte eine kontrollierte Hypothermie nur denjenigen Patient:innen angeboten werden, die längere No-Flow-Zeiten (> 1 min) aufweisen und bei denen das Erreichen der Zieltemperatur innerhalb kurzer Zeit nach Wiedererlangen des Spontankreislaufs realistisch erscheint.

### Anwendung von Temperaturkontrolle bei besonderen Subgruppen

Die gezielte Anwendung von therapeutischer Hypothermie bei spezifischen Subgruppen wird auch durch die Ergebnisse einer kürzlich erschienenen Post-hoc-Analyse gestützt. Nutma et al. analysierten kontinuierliche elektroenzephalographische Messwerte von 479 Patient:innen nach prä- oder innerklinischem Herz-Kreislauf-Stillstand, die eine Temperaturkontrolle mit einer Zieltemperatur von 33 °C oder 36 °C erhalten hatten [20]. Basierend auf den elektroenzephalographischen Befunden 12 h bzw. 24 h nach Herz-Kreislauf-Stillstand wurden die Patient:innen nach dem Schweregrad ihrer enzephalopathischen Veränderungen in eine milde, moderate und schwere Form unterteilt. Unabhängig von der Zieltemperatur wurde bei allen Patient:innen mit schwerer Enzephalopathie ein ungünstiges Outcome (Cerebral Performance Category 3–5) nach sechs Monaten festgestellt. Patient:innen mit milder Enzephalopathie wiesen unabhängig von der gewählten Zieltemperatur überwiegend (88 % vs. 81 %) ein gutes Outcome nach sechs Monaten auf. Demgegenüber zeigte sich ein gutes Outcome bei Patient:innen mit moderater Enzephalopathie, die mit einer Zieltemperatur von 33 °C behandelt worden waren, im Vergleich zu Studienteilnehmer:innen der 36 °C-Gruppe. Diese Ergebnisse weisen darauf hin, dass insbesondere Patient:innen mit einer substanziellen neuronalen Schädigung von einer neuroprotektiven Therapie profitieren können, während Patient:innen mit *per se* günstigen Prädiktoren (milde Enzephalopathie bereits nach 12 h bzw. 24 h) keinen zusätzlichen Benefit durch eine niedrige Zieltemperatur erfahren.

Zu einem ähnlichen Ergebnis kommen auch die Autor:innen einer japanischen Registeranalyse [17]. 1111 Patient:innen nach außerklinischem Herz-Kreislauf-Stillstand wurden hierbei nach dem rCAST-Score^2^ in drei Schweregrade, niedrig, moderat und hoch, des Postreanimationssyndroms unterteilt. Patient:innen wurden entweder mit einer Zieltemperatur von 33–34 °C oder 35–36 °C behandelt. Dabei zeigte sich ein besseres neurologisches Outcome nach 30 Tagen bei Patient:innen mit moderatem Schweregrad und niedrigerer Zieltemperatur im Vergleich zur 35–36 °C-Gruppe. Im Gegensatz dazu wurde kein Unterschied zwischen den beiden Temperaturregimes hinsichtlich des neurologischen Outcome bei Patient:innen mit niedrigem und hohem Schweregrad des Postreanimationssyndroms ermittelt.

### Nach TTM2 – Wie kann eine konsequente Fieberprävention funktionieren?

Die aktuellen ERC-ESICM-Leitlinien empfehlen eine Fieberprävention gegenüber der Anwendung einer Temperaturkontrolle mit 32–36 °C vor dem Hintergrund der Ressourcenschonung und potenzieller unerwünschter Wirkungen durch eine Hypothermie [19]. Hierbei sollte berücksichtigt werden, dass eine konsequente Fiebervermeidung unter Umständen einen höheren Personalaufwand als eine kontrollierte Normo- oder Hypothermie erfordert – einerseits bedingt durch die erforderliche Vigilanz hinsichtlich eines Temperaturanstiegs über 37,7 °C, andererseits durch den häufig notwendigen Einsatz von physikalischen Maßnahmen, zusätzlich zu antipyretischen Substanzen, wie zum Beispiel Kaltwaschungen, Kühlpackungen oder Anbringen kalter Tücher [24]. In der TTM2-Studie erhielten 46 % aller Patient:innen der Kontrollgruppe mit Zieltemperatur ≤ 37,7 °C eine Geräte-basierte Temperaturkontrolle [7]. Es ist davon auszugehen, dass in der klinischen Routine ein ähnlich hoher Anteil an Patient:innen eine Geräte-gestützte Temperaturkontrolle benötigt, um Fieber wirklich zu vermeiden. Mit einem höheren Personalaufwand durch physikalische Maßnahmen, antipyretische Medikation und erhöhte Vigilanz einerseits und ggf. die Notwendigkeit einer Eskalation auf ein Geräte-gestütztes Temperaturmanagement andererseits ist ein erheblicher ressourcenschonender Aspekt von Fieberprävention fraglich und sollte systematisch im Rahmen von Kosteneffizienzstudien erfasst werden.

Nach Publikation der TTM-Studie 2013 weisen Ergebnisse aus Beobachtungsstudien darauf hin, dass ein Verlassen des Hypothermie-Konzeptes zu einer geringeren Sensibilität im Hinblick auf Fieber geführt hat [4, 5]. Dabei wurden auch Auswirkungen auf das Outcome von einzelnen Zentren berichtet [5]. Es ist zu befürchten, dass die aktuelle Empfehlung gegen eine kontrollierte Hypothermie zwischen 32 und 36 °C und Bevorzugung einer Fieberprävention zu einem ähnlichen, wenn nicht noch stärkeren ungünstigen Effekt führen könnte. Vor diesem Hintergrund wird auch in den aktualisierten Leitlinien die Bedeutung einer konsequenten Temperaturkontrolle, basierend auf lokal etablierten Protokollen, betont.

## Fazit für die Praxis

- Die aktuelle Evidenz zeigt keinen Vorteil einer therapeutischen Hypothermie gegenüber einer Fieberprävention bei Patient:innen nach außer- oder innerklinischem Herz-Kreislauf-Stillstand. Aufgrund der besonderen Charakteristika der untersuchten Studienpopulationen sind eine kritische Interpretation und Schlussfolgerung notwendig.
- Ergebnisse, auf denen die ERC-ESICM-Leitlinien 2022 basieren, sind nur eingeschränkt repräsentativ für den klinischen Alltag in vielen Ländern, insbesondere solche mit niedriger Laienreanimationsquote.
- Die aktuellen Leitlinien überlassen dem/r Anwender:in eine hohe Spannbreite im Bereich der zu wählenden Zieltemperatur. Vor diesem Hintergrund gewinnt die Etablierung von lokalen Protokollen zur therapeutischen Temperaturkontrolle an Bedeutung.
- Für die Zukunft erscheint die Untersuchung von speziellen Subgruppen, insbesondere jener mit moderater Enzephalopathie, zielführend und könnte eine differenzierte Applikation von therapeutischer Temperaturkontrolle erforderlich machen.

## Footnotes

1 European Society of Intensive Care Medicine.

2 Post-Cardiac Arrest Syndrome for Therapeutic Hypothermia (rCAST): Risikoklassifikation für Postreanimationssyndrom unter Berücksichtigung folgender Parameter: initialer Rhythmus, beobachteter Herz-Kreislauf-Stillstand/Zeit bis zum Wiedererlangen des Spontankreislaufs, pH-Wert und Lactatkonzentration bei Krankenhausaufnahme, motorische Komponente der Glasgow-Koma-Skala.
